# Shortcut Model for Batch Preferential Crystallization Coupled with Racemization for Conglomerate-Forming Chiral Systems

**DOI:** 10.1021/acs.cgd.1c01473

**Published:** 2022-06-06

**Authors:** Shashank Bhandari, Thiane Carneiro, Heike Lorenz, Andreas Seidel-Morgenstern

**Affiliations:** † Max Planck Institute for Dynamics of Complex Technical Systems, Sandtorstrasse 1, 39106 Magdeburg, Germany; ‡ Otto von Guericke University Magdeburg, Universitätsplatz 2, 39106 Magdeburg, Germany

## Abstract

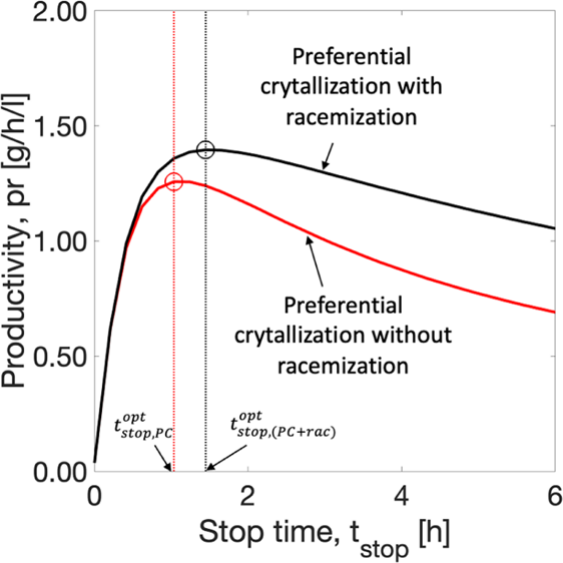

Kinetically controlled preferential crystallization (PC) is a well-established elegant concept to separate mixtures of enantiomers of conglomerate-forming systems. Based on a smaller number of laboratory investigations, the key parameters of an available shortcut model (SCM) can be estimated, allowing for a rapid and reliable process design. This paper addresses a severe limitation of the method, namely, the limitation of the yield to 50%. In order to exploit the valuable counter enantiomer, the crystallization process is studied, coupled with a racemization reaction and a recycling step. It will be shown that the process integration can be performed in various ways. To quantify the different options in a unified manner and to provide a more general design concept, the SCM of PC is extended to include a kinetic model for the enzymatically catalyzed reaction. For illustration, model parameters are used, which characterize the resolution of the enantiomers of asparagine monohydrate and the racemization rate using an amino acid racemase. The theoretical study highlights the importance of exploiting the best stop time for batch operations in order to achieve the highest process productivity.

## Abstract 2

A theoretical investigation to compare the performance of batchwise preferential crystallization with and without a recycling step to an enzymatically catalyzed racemization reactor by using a shortcut model exploiting both crystallization and racemization kinetics.

## 1 Introduction

The two enantiomers of the same chiral molecules often exhibit vastly different effects on biological organisms.^1^ In many cases, only one enantiomer may have a desired physiological effect, whereas the other enantiomer can cause toxicity or have no effect.^2^ Hence, the efficient production and separation of pure enantiomers remains a critical challenge, particularly in pharmaceutical industries.^3^ Enantiomerically pure products can be obtained either by the asymmetric synthesis of only one enantiomer or by the resolution of racemates using separation techniques. Despite remarkable breakthroughs in the field of enantioselective synthesis, it fails to provide a general solution to the growing demands for enantiopure substances in industries.^4^ To address this issue, a variety of separation techniques are used in industries for the resolution of racemic mixtures, for instance, chromatography,^5−7^ membrane processes,^8,9^ and crystallization.^10,11^ The product in the first two processes is obtained in the liquid phase, which often requires crystallization to deliver the final solid enantiopure substance.^12^ It nominates the crystallization process as a potentially more practical technique to directly deliver pure enantiomerically active solid products. The separation of racemic mixtures using direct crystallization is only achievable when each crystal formed is enantiomerically pure and the corresponding physical mixture is called a conglomerate. Unfortunately, the pairs of enantiomers belonging to conglomerate-forming systems are in the minority. The remaining mixtures of enantiomers form either racemic compounds or solid solutions and are more difficult to resolve.^13^

Another process alternative suitable to separate enantiomers is by solid-state deracemization. It can be achieved by grinding^14−16^ or temperature cycling.^17−19^ One of the novel techniques that has gained significant attention in the past decade is Viedma ripening. Contrary to preferential crystallization (PC), it operates near thermodynamic equilibrium and the initially racemic solid phase is converted to an enantiopure form. This process involves several phenomena: attrition, agglomeration, Ostwald ripening, and racemization reaction in the liquid phase. Nevertheless, for it to work on thermodynamic equilibrium, the ripening process might be attractive for some applications, and the choice of method is strongly dependent on the specific features (physical and chemical data) of the compound considered.

In this work, we will consider only the direct crystallization of enantiomers that form conglomerates. For such systems, direct PC is an attractive, cost-effective technique.^11,20^ It is a kinetically driven process that involves selectively growing pure seeds of the target enantiomer from a supersaturated solution while the crystallization of the counter enantiomer is inhibited for a certain period of time by operating in a metastable zone. The general principle, possible process variants, and a shortcut design model will be described below. In any case, it is a clear limitation of using classical direct resolution via PC that the reachable yield is limited to 50%. Thus, it is of great interest to exploit the typically less desired counter enantiomer.

An attractive possibility of using the counter enantiomer is to racemize it to generate up to 50:50 mixtures, which then can be returned to the separation process. This allows avoiding any loss and, thus, achieving the maximum process yield of 100%. Racemization can be catalyzed chemically and enzymatically.^21,22^ The employment of biocatalysis has the following advantages: (i) the use of mild conditions of temperature and solvent, more likely to be compatible with the resolution process; and (ii) the possibility of using enzymes in either free form, a rather less expensive preparation, or immobilized form, which facilitates stabilization, re-use, and separation from reaction media. The rate of racemization is driven by the difference in the concentration between the enantiomers that is generated in our case via PC. It was shown that the occurrence of a racemization reaction improves the supersaturation constellation for PC by accelerating the growth of the target enantiomer and suppressing the nucleation of the counter enantiomer.^23−29^ The concept of coupling the two processes offers a broader range of configurations, which will be discussed below.

The paper is organized as follows. In the next section, we will describe the concept of the process of PC and a shortcut model (SCM) capable of describing its main features. Then, we will introduce several process options for the coupling of racemization with PC. Afterward, a rate model for racemization is presented. This is then incorporated into the SCM for specific coupling configurations. Subsequently, available parameters of the SCM including racemization are presented for a specific case study, namely the enantiomers of asparagine monohydrate (dl-Asn) in water. General recommendations are finally provided based on comparing PC with and without racemization process performance indicators such as productivity and yield.

## 2 Batchwise PC

### 2.1 Principle and Variants of Operation

PC starts with the provision of an undersaturated racemic feed solution, which is transformed through cooling into a slightly supersaturated metastable solution. After seeding with the target enantiomer, this kinetically driven resolution process is triggered, during which the seeds grow, while the nucleation of the counter enantiomer is inhibited.^11^ To implement the process, it is important to know the width of the metastable zone in which PC takes place. The unavoidable nucleation of the counter enantiomer limits the exploitable time window and requires stopping the batch process at a specific stop time.

In recent years, numerous studies on improved variants of PC have been reported, mainly focused on delaying the nucleation of the nontarget enantiomer in two coupled crystallizers,^30−32^ enabling continuous mode in coupled PC,^33,34^ fluidized bed crystallization,^35,36^ or coupling PC with selective dissolution.^37^

### 2.2 Population Balance and SCMs to Describe PC

Population balance models (PBMs) are powerful tools and are used frequently to describe crystallization processes. They provide a comprehensive overview of the evolution of crystal size distribution while accounting for various kinetic mechanisms such as crystal growth, dissolution, nucleation, agglomeration, and attrition. An overview of different applications of the PBM and methods of the solution can be found in the literature.^38−41^ Another solution technique is the method of moments, which describes PC by using at least five differential equations. It can take into account, besides growth, also nucleation and eventually agglomeration and breakage.^42^ There are significant experimental efforts required to fully parametrize the underlying submodels related to the different kinetic phenomena taking place. Thus, there is a need for the development of even simpler models that provide quicker estimates of key performance indicators such as productivity, purity, and yield with relatively easy-to-find parameters. By neglecting nucleation and assuming monodisperse spherical seed crystals, the zeroth moment is a constant and the first, second, and third moments are directly correlated. This offers the opportunity to describe the exploitable productive initial phase of the PC process with a very low number of ordinary differential equation (ODE) as introduced in the paper.

In our previous study, we developed an SCM for the simulation of isothermal batch PC for the conglomerate-forming system. The model is based on quantifying in a simplified manner the “overall mass transfer” between the two phases, assuming a lumped kinetic mechanism for crystal growth and nucleation.^43^ It is briefly summarized below before being extended in Section 3.3 to include various variants of coupling the process with racemization. The main assumptions underlying the SCM for PC are as follows:

- nucleation and growth rates are lumped into a power law to jointly cause liquid-phase mass depletion and solid-phase mass build up.
- All crystals of one enantiomer are spheres of identical increasing size.
- Very small particles of the counter enantiomer below a contamination threshold are assumed to be initially just passively present along with the introduced seeds of the preferred enantiomer.
- As an essential parameter, a stop time *t*_stop_ is introduced to activate “nucleation” and growth of the particles of the undesired counter enantiomer. Beyond this time, the solid-phase contamination starts.
- The total number of crystals at the beginning of the process is equal to the number of crystals at the end of the process.

The mass balance equations of the SCM for a crystallizer (C) used for PC are given in 1. The mass depletion rate of each enantiomer in the liquid phase (eqs 1 and 2) is a result of three factors: an effective crystallization rate *k*^eff^, the total surface area of all the crystals, with *N*_*i*_ being the total number of spherical particles of radius *R*_*i*_, and the driving force term, which is a function of supersaturation *S*_*i*_ and the effective order of crystallization *n*^eff^.
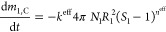
1
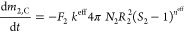
2

An additional parameter *F*_2_ is activated when counter enantiomer contamination starts at the stop time *t*_stop_ (eq 3). This time terminates the production period.
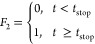
3

A mass balance for the solvent is included (eq 4) to consider the option that enantiomers can form solvates as the crystalline phase. This requires including the ratio of the molecular masses of the solid solvate, *M*_solvate_, and the nonsolvated enantiomers, *M*_*i*_.
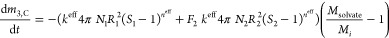
4

The mass balances of the solid phase (superscript S) balances counter-balances for the enantiomers the corresponding mass depletions in the liquid phase (eqs 5 and 6).
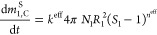
5
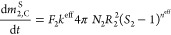
6

The radii of the growing spherical particles of each enantiomer can be calculated from the total solid masses and the number of particles using eq 7.
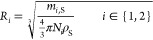
7

The calculation of the composition-dependent supersaturation *S*_*i*_ (eq 8) is essential to quantify the process transient.
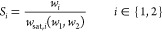
8

Hereby, *S*_*i*_ is defined as the ratio between the mass fractions (eq 9) at the current state and the mass fractions at the equilibrium state.
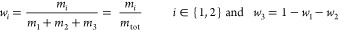
9

The composition-dependent calculation is performed by exploiting the ternary phase diagram of the specific chiral system.^4,43^ Current state compositions are evaluated using geometrical considerations and applying cartesian coordinates *X* and *Y*, which are connected with the corresponding mass fractions. The following transformation rules hold^29^
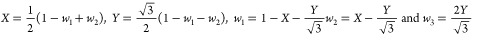


The above introduced equations of the SCM for PC can be solved simultaneously after providing the following initial conditions for the case that enantiomer 1 is the target: *m*_1_^0^, *m*_2_^0^, *m*_3_^0^, *m*_1_^S,0^, *R*_1_^0^, *N*_1_^0^, *R*_2_^0^, *N*_2_^0^, *w*_sat,1_^0^, and *w*_sat,2_^0^.

The three free parameters of the SCM required to solve eqs 1–9, namely *k*^eff^, *n*^eff^, and *t*_stop_, are specific to each case study. They need to be supplied based on the results of a relatively small number of dedicated preliminary experiments, as described and illustrated in ref (43).

## 3 Coupling PC and Racemization

This paper represents an application of the already published SCM. In our previous study, we have described all the essential features of SCM that we have directly applied in this work. A detailed explanation of assumptions, such as the applicability of a stop time (*t*_stop_) and an effective crystallization rate constant (*k*^eff^) (lumping of nucleation and growth), can be found in our previous work.^43^

### 3.1 Possible Coupled Process Schemes

There are various ways in which the racemization step can be integrated with PC. In this work, two setup schemes are studied: a spatially integrated and a spatially segregated process. In a spatially integrated process, racemization and crystallization take place in the same reactor. In the second process scheme, racemization and crystallization are performed in separate units. The racemization reaction must be carried out by a racemizing agent. As mentioned above, our focus in this work is on the application of biocatalysis to improve PC.

There is the possibility to apply homogeneous catalysis using free soluble enzymes and heterogeneous catalysis with an immobilized enzyme. Furthermore, there are the options of providing the enzyme within the vessel in which the crystallization is performed or in a separate vessel.

**Figure 1:**
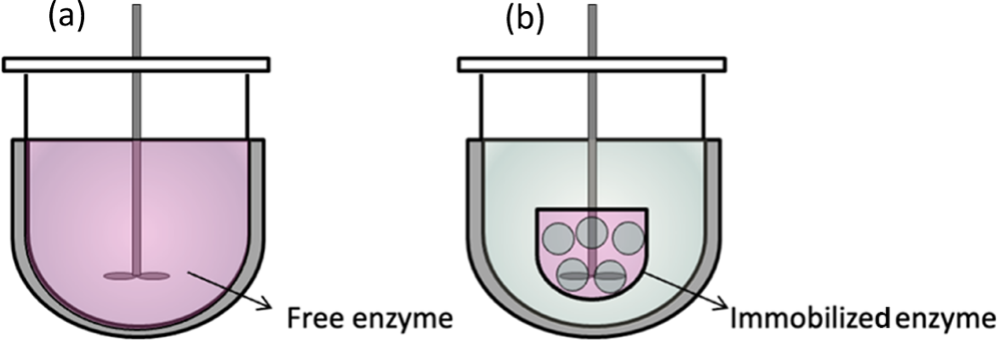
Illustration of PC combined with racemization taking place simultaneously in the same well-mixed vessel. (a) With a free (dissolved) enzyme acting as a racemization catalyst. (b) With an immobilized enzyme fixed in a basket which can be mounted to the stirrer.

Two options of having the free or immobilized enzymes in the crystallizer are illustrated in Figure 1. This is attractive considering the amount of equipment required. In contrast, there are obviously the disadvantages of more complicated downstream processing required to separate at the end of the batch the valuable enzyme from the mother liquor and the fact that the operating temperature of racemization is bound to the crystallization temperature.

**Figure 2:**
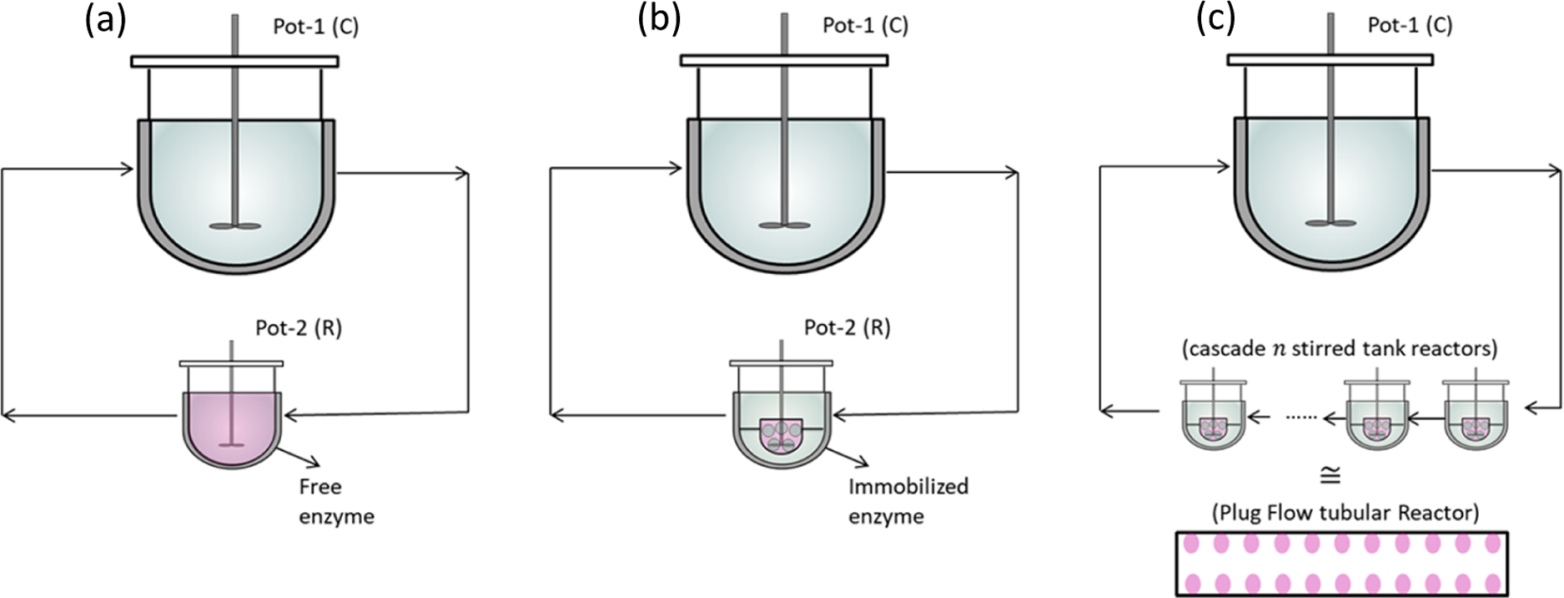
Illustration of PC performed in a well-mixed vessel combined with racemization taking place separately in a different vessel. (a) Racemization with a free enzyme in a well-mixed (stirred tank) reactor. (b) Racemization with an immobilized enzyme in an STR. (c) Racemization with an immobilized enzyme in a tubular reactor corresponding to a cascade of STRs.

Options for spatially segregating are shown in Figure 2. The standard vessel for PC is combined with an external racemization unit via a recycling loop. Three types of racemization reactors are studied: a single stirred tank reactor (STR) with free enzyme, an STR with an immobilized enzyme, and a tubular fixed-bed reactor, which can be represented by a cascade of STRs. The racemization units can be operated at a higher temperature than PC to speed up the reaction rate and to avoid crystallization in the connections outside of the crystallizer.

Model equations capable of describing all the reactor types shown in Figures 1 and 2 will be introduced in Section 3.3 after providing in the next section a model for describing the rates of enzymatic racemization.

### 3.2 Quantifying the Rate of Racemization Reactions

In order to quantify the coupled schemes shown in Figures 1 and 2, an additional submodel is needed which describes the rate of the racemization reaction of the two enantiomers (d, l or 1, 2), that is, d⇌l. This reaction changes in the presence of the catalyst (e.g., a racemase) the liquid-phase composition. It approaches an equimolar (racemic) equilibrium composition.

A simple way to describe the forward and backward reactions is to apply first-order rate models. However, in the case of applying enzymes, the actual reaction mechanism is more complex. It includes the formation of intermediate complexes between both reactants and products with the biocatalyst. In addition, enzymatic reactions exhibit saturation at high substrate concentrations relative to the amount of catalyst, and they may display suboptimal binding, leading to inhibited kinetics.^21^Equation 10 describes the rate of forming a preferred enantiomer (here 1) and the corresponding rate of consuming the counter enantiomer using a racemase.^44^
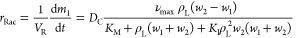
10

In this equation, *V*_R_ is the reaction volume used for scaling, and *D*_c_ is the dosage or concentration of the catalyst expressed in mg enzyme/L or g support/L for the free and immobilized enzyme, respectively. Furthermore, *v*_max_ and *K*_M_ are parameters characteristic of a specific enzyme under given conditions, such as the reactant and temperature range. The third enzyme-related parameter *K*_I_ can be applied in case an inhibition occurs, which causes an alteration in the catalytic action of the enzyme. Finally, ρ_L_ is the density of the liquid phase.

Properly parametrized eq 10 can be applied to describe the rates for both free and immobilized enzymes.

### 3.3 Extension of the SCM for Different Variants of Combining PC and Racemization

#### 3.3.1 Process Integration in the Same Vessel

The combination of PC with racemization taking place in the same vessel (see Figure 1) can be quantified by extending the SCM of PC. The following assumptions are taken into account in formulating the following equations:

- The racemization unit is of the stirred tank type.
- The reactor has a constant temperature and volume.
- Perfect mixing of the enzyme in the reaction volume.
- The presence of the enzyme has no effect on the solubility of the enantiomers.

The changes in mass of the preferred enantiomer in the liquid phase in the crystallizer–reactor (C + R) are due to the combined effects of crystallization and racemization as given by eq 10. Similarly, eqs 11 and 12 describe the changes in the mass in the liquid phase of the counter enantiomer and the solvent, respectively. The rate of change of solid mass is exclusively caused by crystallization. Therefore, the mass balance for the solid phase remains the same as described in eqs 5 and 6 with the stoichiometric coefficients (ν_*i*_ = −1 or 1) required in the reaction term as follows
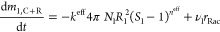
11
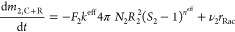
12

Both the applications of a free or an immobilized enzyme can be described if the correct eq 10 is used for the rate of reaction.

#### 3.3.2 Spatially Segregated Processes of PC and Racemization

##### 3.3.2.1 Racemization in a Well-Mixed Reactor

In a spatially segregated system, batch PC is connected with an external enzymatic reactor by a recycling loop of the liquid phase. In this study, two types of reactor design were investigated for the enzymatic reactor: a single STR and a cascade of *n* STRs. Similarly to the process scheme described in the previous section, the SCM is extended to be able to describe both spatially segregated systems. As depicted in Figure 2, a solid free recycle stream passes through the racemization reactor for a specific residence time. It returns to the crystallizer with a liquid phase enriched in the preferred enantiomer. The following assumptions are used for designing the process:

- The crystallization step is modeled as a stirred tank type, whereas the enzymatic reactor is modeled as either a single stirred tank or a cascade of stirred tanks.
- The mass flow rate from all the units is kept constant.
- All the streams are crystal free and enzyme free.
- Perfect mixing of the free enzyme in the reaction volume.

In this setup, the modified rate equations of the crystallizer must include the input and output flow, as expressed in eqs 13–16
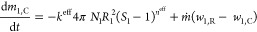
13
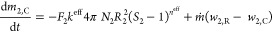
14
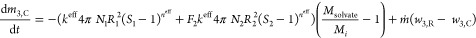
15where *ṁ* is the mass flow rate, expressed in g/h, and variables *w*_1,R_ and *w*_1,C_ are the mass fraction compositions of the streams leaving the reactor and the crystallizer, respectively.

The liquid phase depleted in the preferred enantiomer passes through the enzymatic reactor. Considering the reaction kinetics described in eq 10, the resultant mass balance equations for a single stirred tank racemization reactor are given by eqs 16–18.
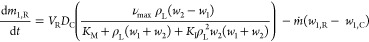
16
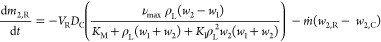
17
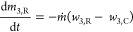
18

##### 3.3.2.2 Racemization in a Cascade of Well-Mixed Reactors

**Figure 3:**
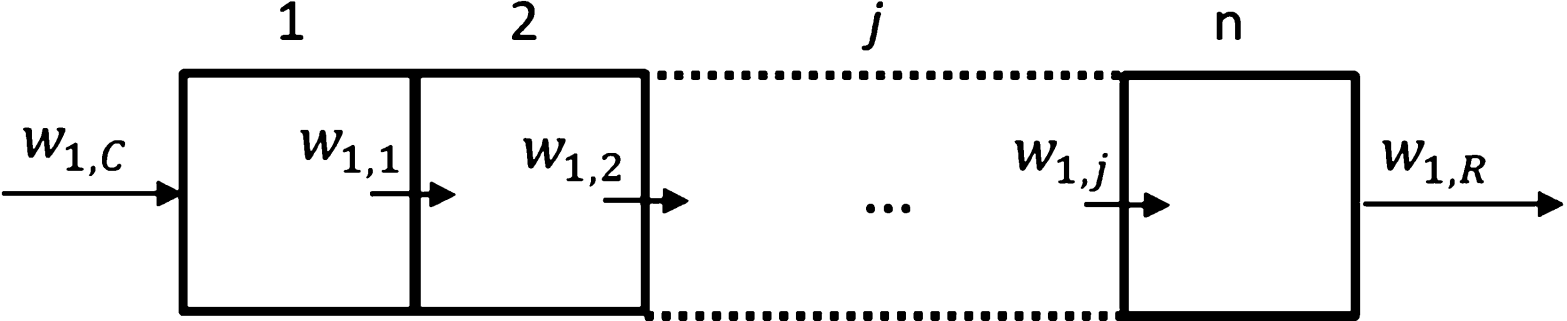
Diagram to represent the flow of a selected enantiomer 1 in a cascade of *n* STRs connected in series (see Figure 2c).

In a cascade of *n* STRs, the recycle stream from the crystallizer is passed through *n* identical reactors in series (*j* = 1, 2, ..., *n*) with a total volume equal to that of a single tank. The inlet concentration of the liquid phase at the first reactor is equal to the outlet concentration of the crystallizer (*w*_1,0_ = *w*_1,C_). The cascade of racemizing reactors produces a stream enriched in the preferred enantiomer, which is recycled back to the crystallizer. The mass balance over *n* reactors as illustrated in Figure 3 is given for enantiomer 1 by eqs 19–24.
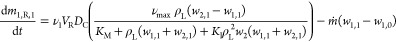
19
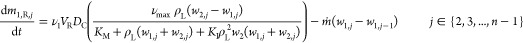
20
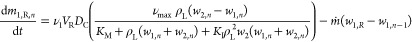
21

Besides, similar equations for enantiomer 2 hold the solvent balance
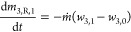
22
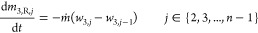
23
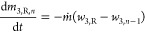
24

The systems of differential equations given above and corresponding to both spatially integrated and spatially segregated separation–reaction systems were solved using MATLAB.^45^ Because of the various magnitudes of time constants involved, the reliable solver “ODE15s” was used because it is capable of solving stiff sets of equations.

### 3.4 Criteria to Evaluate Process Performance

In order to estimate the kinetic parameters for the SCM, experimental results giving information about the progress of PC are required. For this purpose, a polarimeter is used to capture online measurements of the optical rotation angle (α) during the process. The changes in concentration in the mother liquor generated by crystallization result in changes in the optical rotation as the following
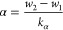
25where, α is the optical rotation and *k*_α_ is a component-dependent calibration parameter dependent on temperature. The optical rotation can also be quantified as a function of the total solute concentration and the enantiomeric excess of the liquid phase (ee_L_) as follows
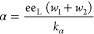
26

The ee_L_ is calculated from the mass fraction of the enantiomers according to eq 27.
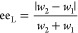
27

In a classical batch PC process, the ee_L_ is zero at the beginning of the process because the liquid phase is racemic. Then, it increases until it reaches a maximum and depletes following the crystallization of the other component.

One of the attractive features of the SCM is the ability to quickly access key design parameters, for instance, productivity, purity, and yield, to estimate process efficiency. They are essential for evaluating performance during the process design and comparing the process with different alternative processes.

Purity as a key requirement in enantioseparation can be defined as the mass of the target enantiomer crystallized over the total solid mass produced during the batch time. It is expressed as follows
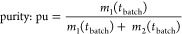
28

If the batch time is shortened by the stop time *t*_stop_, the model predicts a purity of one. To compare different process options, it is attractive to compare the achievable productivities. Productivity can be defined as the mass of the target enantiomer produced in a batch time per unit volume, which is given by the following expression
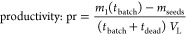
29where *m*_1_(*t*_batch_) is the mass of the target enantiomer available at the end of the batch, *m*_seeds_ is the initial mass of seeds, and *t*_dead_ is the extra time needed for preparation and cleaning between batches. For normalization of the liquid phase volume in the crystallizer, *V*_L_ is used for both cases (PC alone and combined with racemization).

Yield can be defined as the mass of the target enantiomer produced during a batch time over the maximum theoretical product mass (*m*_max_^0^) that can be achieved. It is given as follows
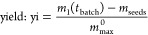
30

The maximum theoretical product *m*_max_^0^ depends on the solubility of the racemic mixture at the initial and saturation states. It is expressed as follows 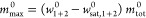
31

## 4 Model Parameters Based on a Case Study

The model system evaluated in this theoretical study was asparagine monohydrate in water. For this system, there is a large set of experimental data available. The L-enantiomer was considered as the target molecule. The model compound is a representative of a conglomerate-forming system for which PC can be applied to resolve a racemic mixture.

The model enzyme used is an amino acid racemase (AAR). The reaction kinetics for racemization in free soluble and immobilized fashion were reported in ref (24). The application of the AAR was also demonstrated at operating conditions of PC. The AAR from *Pseudomonas putida* was immobilized on the commercial support Purolite ECR 8309. The immobilized racemase has been successfully applied as the racemization agent in temperature cycling deracemization,^19^ and it has been investigated for its application to improve enantioselective chromatography.^26^ The kinetic data necessary to model an enzymatic reactor were obtained experimentally for both soluble and immobilized preparations.^24^

The physicochemical parameters used in the simulations were described in our previous publications,^24,43^ and they are shown in Tables 1 and 2.

**Table 1:** SCM Parameters for d-/l-asparagine Monohydrate in Water (Estimated at *T*_cryst_ = 30 °C, i.e., for *S*^0^ = 1.24)^a^

parameter	symbols	value	unit
stop time	*t*_stop_	3.14	h
effective order of crystallization kinetics	*n*^eff^	6.10	
effective crystallization rate constant	*k*^eff^	62.3	g h^–1^ cm^–2^

a More data are given in ref (43).

**Table 2:** Kinetic Parameters of Asparagine Racemization Using the Free and Immobilized (Equation 10) AAR^24^^a^

type	*T* [°C]	ν_max_ [10^2^ g h^–1^ mg enzyme^–1^]	*K*_M_ [10^2^ g mL^–1^]	*K*_I_ 1/[10^2^ g mL^–1^]	*D*_C_ [mg enzyme mL^–1^]
free	30	18	0.6	0.3	30
free	40	24	0.3	0.1	30
immobilized	40	30^b^	2.6	0	30^c^

a The immobilized material was prepared with a enzyme load of 35 mg enzyme/g support.

b Calculated from enzyme load on the immobilization support, corresponds to ν_max_ = 1038 [10^2^ g h^–1^g support^–1^].

c Calculated from the characteristics of the column packing, corresponds to *D*_C_ = 0.9 [g support mL^–1^].

**Figure 4:**
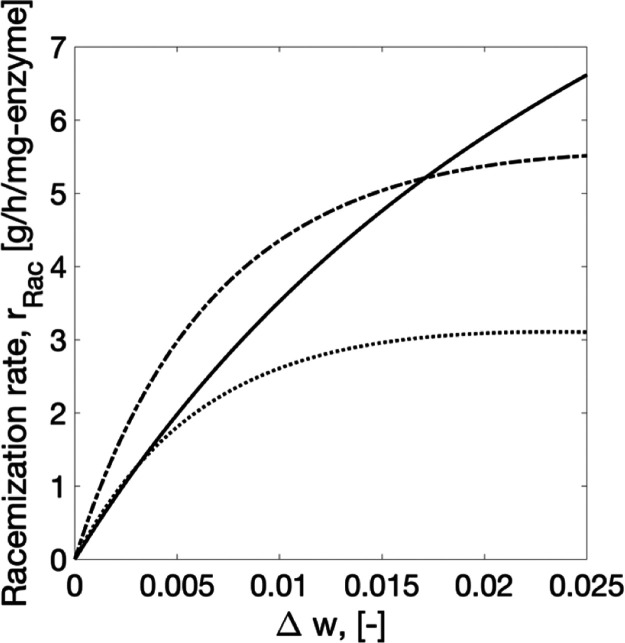
Racemization rate of the free (dashed curve) and immobilized (solid curve) AAR at 40 °C, and the free (dotted curve) AAR at 30 °C as a function of enantiomeric excess is shown. Comparison is performed by keeping the same reactor volume and dosage for all three cases. Parameters of eq 10 from Table 2.

The free AAR is affected by inhibition effects.^46^ They are accounted for by the constant *K*_I_ (Table 2). At a higher initial substrate concentration, the free AAR kinetic profile reaches a maximum value of the reaction rate before dropping (Figure 4). Immobilization of the AAR results in an apparent lower affinity for the substrate, as observed by the increase in *K*_M_ (Table 2), and no effect of substrate inhibition. Both results are likely a consequence of altered concentration profiles caused by mass transport processes in the porous support.^47,48^ At the highest concentrations and driving forces investigated experimentally, the immobilized enzyme (the solid curve in Figure 4) even offers faster reaction rates than the free preparation (dashed and dotted curves in Figure 4).

The density of liquid, ρ_L_ is given for the asparagine monohydrate/water system as follows.^44^
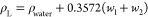
32
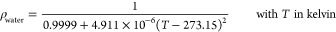
33

For the studied AAR in its free form, the presence of high concentrations of reactants causes a decrease in the reaction rate.^24^ This is included in eq 10 by activating the parameter *K*_I_.

## 5 Evaluating the Potential of Integrating Racemization

### 5.1 Comparison between Single PC and PC Spatially Integrated with Racemization

**Figure 5:**
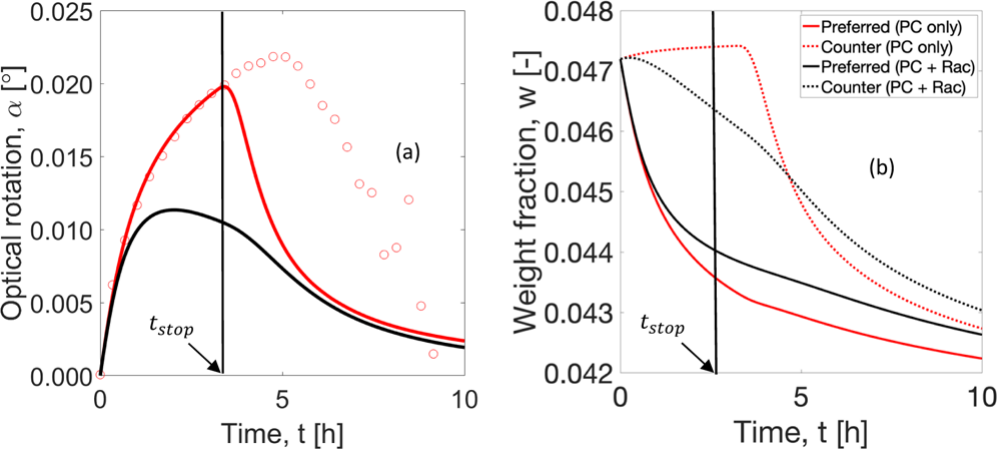
Comparison between experimental results of batch PC and SCM simulations for dl-asparagine monohydrate in water. Evolution of (a) optical rotation and (b) weight fractions of target and counter enantiomers in the liquid phase. Red circles: experimental profile for conditions mentioned in Table 3. Red: SCM results without racemization. Black: SCM results with in situ racemization. *t*_stop_ = 3.14 h indicates the limits of the SCM simulation validity.

In this section, the simulations of coupled PC and racemization performed in the same vessel using the free enzyme (Figure 1a) are compared with experimental and theoretical results of single PC from our previous publication.^43^ The process conditions and solubility information used during experiments and SCM simulations of single PC are given in Tables 1 and 3. The curves in Figure 5a show the resulting optical rotation profiles. The SCM simulations (solid red curves) provide a good agreement with the experimental results (red circles) until the stop time. After this period, crystallization of the counter enantiomer takes place and the purity of the solid phase drops. Therefore, for a strong product purity constraint of 100%, the process is applicable only until the stop time is reached. To improve the performance, an integrated racemization step is now considered jointly with the PC process (Figure 5, black curves). The reaction reduces the difference in concentrations of the two enantiomers in the liquid phase by converting the antipode into the target enantiomer. As a result, the maximum optical rotation achieved is lower than in the process without racemization (Figure 5a). The process combination also keeps the system closer for a longer period to the lower boundaries of the MZW, which delays the nucleation of the nontarget enantiomer. To allow for a theoretical comparison, the same stop time applicable for single PC was used to simulate the combination of PC with racemization. However, the presence of racemization has an impact on the stop time. A longer operation can be exploited. Thus, using the same stop time is a conservative limiting case. The process performance of the integrated process will benefit from a longer production window, as discussed below.

**Table 3:** Summary of Experimental Conditions for Batch PC of dl-asparagine Monohydrate^43^

parameter	symbols	value	unit
initial mass fraction	*w*_*i*_^0^	4.56	10^–2^ g g^–1^
initial saturation mass fraction	*w*_sat,*i*_^0^	3.68	10^–2^ g g^–1^
initial supersaturation	*S*^0^	1.24	
crystallization temperature	*T*_cryst_	30	°C
saturation temperature	*T*_sat_	35	°C
calibration parameter of polarimeter	*k*_α_	0.048	g g^–1^
seed mass *m*_seed_	*m*_1_^S,0^	0.2	g
volume of crystallizer	*V*_L_	0.2	L

Predicted mass fraction depletions of the two enantiomers in the liquid phase are depicted in Figure 5b. The seeded enantiomer has a higher crystallization rate than its antipode and, therefore, it is characterized by a steeper drop in the concentration. The racemization reaction occurring in the coupled process (solid black curves) causes depletion in the counter enantiomer concentration even before the stop time, for it being converted into the target enantiomer. This effect does not happen in the single batch PC (solid red curves). In addition, the overall concentration of the target enantiomer is kept higher than in the process without racemization. The results indicate that the racemization does not completely block the increase in enantiomeric excess during crystallization. However, there is still a clear benefit in applying the reaction. It allows maintaining elevated levels of supersaturation favorable for crystal growth and, consequently, productivity of the process (eq 29).

### 5.2 Evaluation of Variants of the Spatially Segregated Process

The SCM simulations of the spatially segregated process were performed using two design configurations for the external racemization reactor: a single STR and a cascade of STRs (Figure 2b,c).

#### 5.2.1 Influence of Enzyme Preparation

The combination of PC with a single stirred tank racemization reactor was simulated as explained in Section 3.3.2. The values of the operating parameters required to run the simulations were selected based on the scale of the setup used in our laboratory. The conditions are shown in Table 4.

**Table 4:** Operating Parameters for the Racemization Reactor in the Spatially Segregated Process

parameter	symbols	value	unit
flow rate	*V̇*	3.5	mL/min
reactor volume	*V*_R_	2	mL

**Figure 6:**
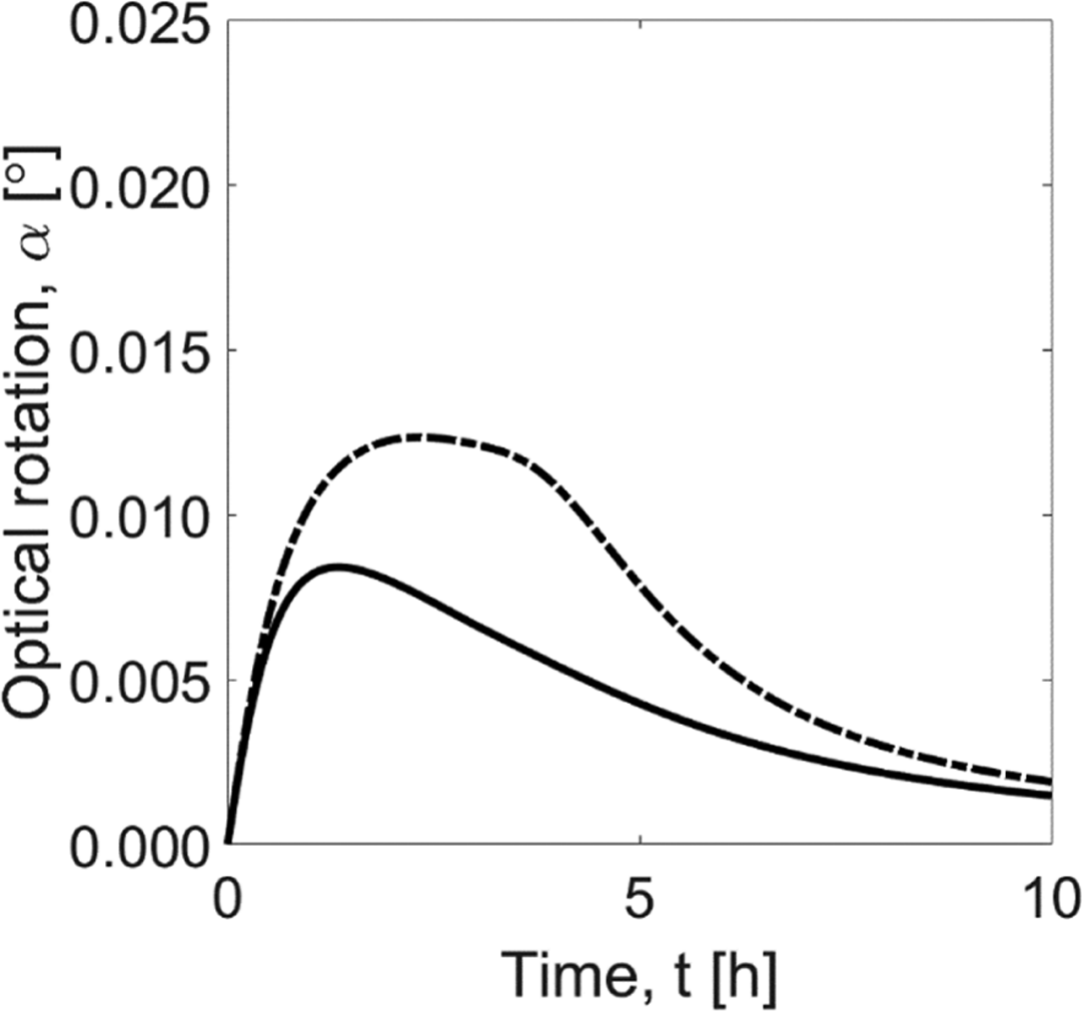
Simulation of optical rotation profiles in a batch crystallizer connected to a single stirred tank enzymatic reactor using free (dashed curve) and immobilized (solid curve) enzymes.

The comparison of the transients predicted by the model for homogenous (Figure 2a) and heterogeneous (Figure 2b) enzymatic catalysis is depicted in Figure 6. The simulated optical rotation profile using free soluble and immobilized enzymes is represented by the dashed and solid curves, respectively. In both reactors, the enzyme dosage *D*_C_ used was the same (see Table 2). The process combination using the immobilized enzyme showed a significantly lower peak of optical rotation. In this configuration, racemization of the counter enantiomer was faster, avoiding higher differences in concentration between the stereoisomers. As mentioned above, the reaction kinetics caused by enzymes change upon immobilization. In the case of the AAR presented in this study, immobilization introduced mass transfer effects but allowed higher reaction rates when increasing reactant concentrations (Figure 4). Unlike the free AAR, the immobilized preparation did not present inhibition effects at conditions of high substrate concentration. Therefore, for the given process, the kinetic behavior of the free enzyme generates a lower rate of racemization than the immobilized preparation (see Figure 6).

Because of the better performance, the immobilized AAR (eq 10, Table 2) is considered in the following process design calculations.

#### 5.2.2 Influence of Size of the Enzymatic Reactor and Flow Rate

An evolution of productivity was performed for the process shown in Figure 2a to identify suitable mean residence times of the liquid phase for a set of reactor volumes *V*_R_ ranging from 0 to 5 mL and flow rates *V̇* ranging from 0 to 5 mL/min (again conditions typical for laboratory scale investigations). The residence time is given by eq 34
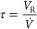
34

**Figure 7:**
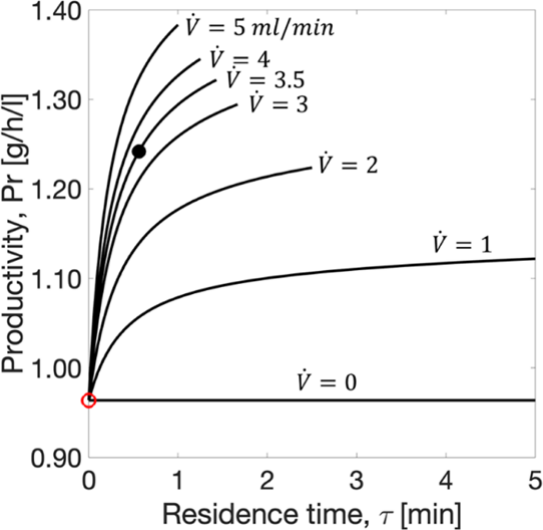
Impact of the residence time (eq 34) on the productivity (eq 29) of PC coupled with heterogeneous enzymatic racemization in a single STR. Black dot: productivity for *V̇* = 3.5 mL/min and *V*_R_ = 2 mL (Table 4). Red circle: productivity for PC without racemization.^43^

The impact of these parameters on productivity can be seen in Figure 7. The boundary condition of no-flow between the units (i.e., *V̇* = 0) represents PC without racemization. At these conditions, the productivity is constant and equals 0.96 g/h/L, as previously reported.^43^ By increasing the flow rate, higher amounts of mother liquor and, consequently, of the reaction substrate are available for racemization at any given instant. This generates an increase in the reaction rate, providing higher productivities. There is nevertheless a limit to that improvement. For a fixed residence time, productivity does not significantly benefit from an increase in the flow rate. In the limiting case, when the flow rate is sufficiently high, that is, *V̇* → ∞, the behavior of the process would shift from a spatially segregated to a spatially integrated process.

The results in Figure 7 also demonstrate that increasing the residence time of the liquid in the reactor leads to higher productivities. For a constant volumetric flow rate, a longer residence time is achieved by using a larger reactor volume. Nevertheless, larger reactors can only improve the resolution process to a certain extent. Increasing the reaction volume implies operating with a higher amount of enzyme, which promotes a faster conversion of the counter enantiomer. Thus, the productivity profiles reach their limiting values at fixed flow rates. In the case of immobilized enzymes, the limiting report between the reactor volume and the catalyst dosage is bound to the characteristics of a packed reactor. The AAR used in this study was immobilized at a load of 35 mg enzyme/g support, and a 2.1 mL column reactor was packed with 0.9 g support/mL (Table 2). Lower amounts of immobilized support per volume are acceptable for the enzyme carrier being dispersed in the liquid phase. If higher amounts of the support are applied, the compression of the packing can compromise the enzymatic activity.

#### 5.2.3 Racemization in a Cascade of Tank Reactors

**Figure 8:**
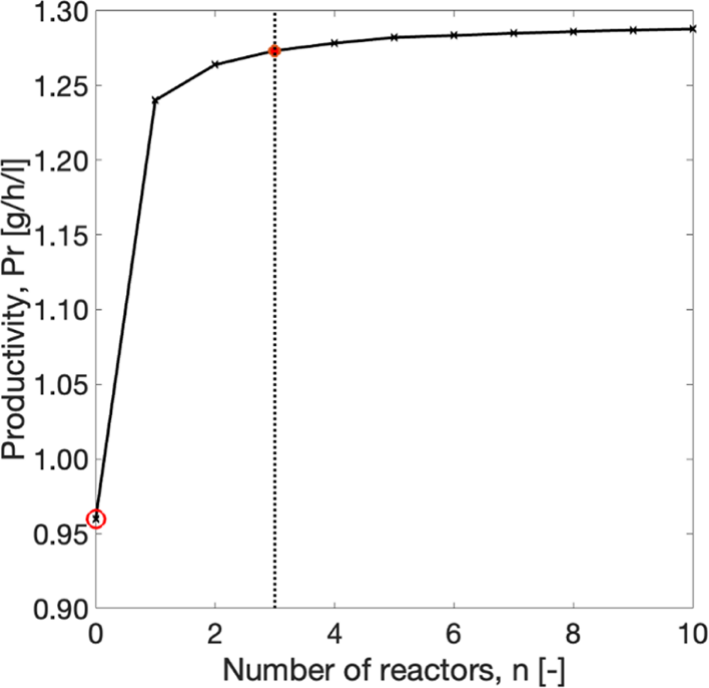
Impact of the number *n* of the STRs on the performance (eq 29) of a crystallizer coupled with a cascade of STRs in series. Red dot: productivity at *n* = 3 (used in further calculations). Red circle: productivity for PC without racemization.^43^

In this configuration, SCM simulations are performed for a cascade of STRs in series connected with the crystallizer (scheme in Figure 2c). All the reactors are assumed to have equal volume and residence time, and the total volume is identical to that of a single STR discussed above (see Table 4). To investigate the impact of the number of reactors on the performance of the process, productivity was estimated at a range of values of n. The results can be seen in Figure 8. Racemization reduces the difference in concentration between the enantiomers while moving to each reactor of the cascade, so the driving force available for the *j*th reactor is lower than that for the following. Hence, the productivity profile reaches a plateau at a relatively high number of reactors. At that range, the increase in productivity may not compensate for the cost of the addition of subsequent reactors. A detailed study to estimate the optimum value of *n* of the spatially segregated process was not intended here. Therefore, it is selected reasonably based on the nature of the plot. A realistic value of *n* = 3 was considered for further calculations. Keeping a constant total reactor volume, the productivity gain from a single reactor to a cascade of three equally sized reactors is almost 3%.

#### 5.2.4 Influence of the Stop Time on Productivity

**Figure 9:**
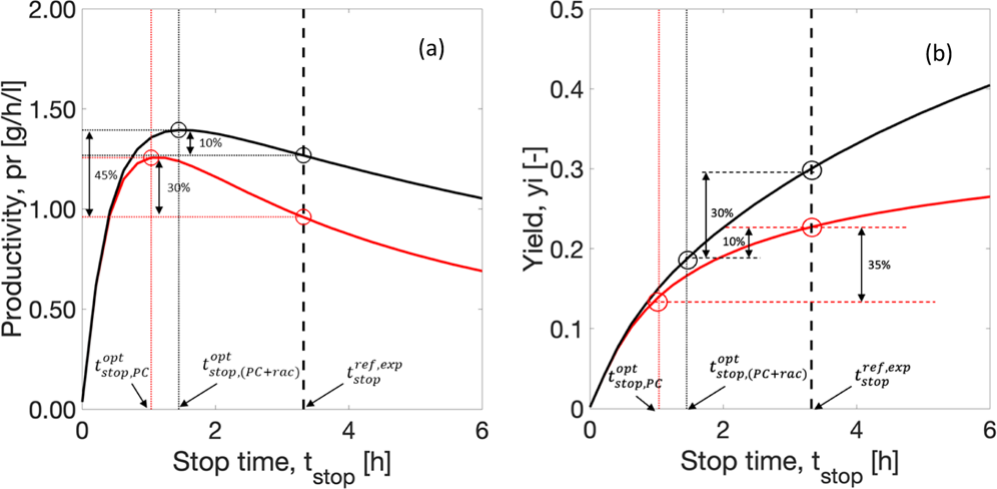
Productivity and yield estimations and the impact of the stop time. Red curve: single batch PC without racemization. Black curve: a spatially segregated process with racemization in a cascade of three STRs. The dashed line marks the stop time determined from experimental data,^43^*t*_stop_^ref, exp^ = 3.14 h. By operating at optimum *t*_stop, PC_^opt^ = 1 h and *t*_stop, (PC+rac)_^opt^ = 1.4 h, the productivity increases by approximately 30 and 10%, and the yield drops by 35 and 30% on single PC and spatially segregated coupling, respectively. Overall productivity increases by 45% and yield drops by 10% compared to the reference case. The simulation conditions are described in Tables 3 and 4.

The time to stop the batch is of the utmost importance. It was defined in the SCM as the time until which the crystallization of the counter enantiomer is negligible. This parameter has been so far estimated based on strict chiral purity constraints on single PC. To achieve higher productivity, it is important to investigate the optimum moment to stop the process. As a change in the stop time directly impacts the yield of the process, it is therefore critical to investigate the overall yield of the process. It is clear that, by coupling PC with racemization, the applicable stop time can be extended. In this section, we have investigated the impact of a range of stop times on the productivity and yield of the process. As defined earlier, for a constant volume, productivity (eq 29) is directly proportional to the product mass collected and inversely proportional to the stop time. It rises as the product mass collected reaches a maximum and depletes again with an increase in the stop time. Yield (eq 30) is the ratio of the total product mass to the maximum theoretical product mass that can be achieved. It rises with an increase in the product mass collected and becomes flat at equilibrium. The red curve in Figure 9 represents the process without racemization. At the stop time determined with 100% purity based on experimental data, that is, *t*_stop_^ref,exp^ = 3.14 h, considered as reference, the estimated productivity was 0.96 g/h/L, as mentioned above. However, the simulations showed a maximum productivity at an earlier process time, *t*_stop, PC_^opt^ = 1 h. If the process is interrupted then, an improvement of around 30% in productivity can be achieved (Figure 9a). However, the yield in that case drops by approximately 35% due to stopping the process earlier (Figure 9b). The black curve in Figure 9 is the resulting simulation of a spatially segregated process with three STRs (scheme in Figure 2c). It also shows an optimal productivity value and corresponding yield value before the stop time determined for PC without racemization. By operating the coupled process until the earlier time at *t*_stop, (PC+rac)_^opt^ = 1.4 h for maximum Pr, the productivity can be improved by around 10%. Nevertheless, it deteriorates the overall yield by around 30%. This is an interesting result because the racemization step acts by avoiding crystallization of the counter enantiomer and making the process more robust for a longer period. Overall, productivity can be improved by 45% and yield deteriorated by 10% with the application of shorter stop time and integration of racemization compared to single PC without racemization.

## 6 Conclusions

Different options for incorporating an enzymatic racemization step into a PC process were evaluated theoretically using a shortcut model (SCM). The parameters used hold for the resolution of a pair of two enantiomers of an amino acid and the racemization kinetics for an AAR. The SCM exploits a rough estimation of the exploitable maximum duration of the kinetically controlled PC process and allows a rapid prediction of the productivity of the overall process. With respect to enzyme provision, two options were compared. For the separation problem considered, the model predicts that at higher substrate concentrations, the immobilized enzyme performs better than the free enzyme. It was further demonstrated that the core model can be easily extended to quantify possible gains if a cascade of consecutive tank crystallizers is used instead of conventional single tank operation. The comparison given regarding process performance and ranking is just valid for the example considered. Because of the wide range of possible nucleation rates, growth rates, and enzyme specific racemization rates, it is not yet possible to generalize these results. The conceptual approach presented can be extended to treat other configurations of combining crystallization and racemization processes, including regimes including recycling of the mother liquor obtained at the end of one batch to a new one and continuous operation.

## Notation2-col

*D*_C_: dosage or concentration of the catalyst [mg enzyme L^–1^]
ee_L_: enantiomeric excess of the liquid phase [-]
*F*_2_: counter enantiomer contamination factor [-]
*i*: index of target and counter enantiomers (1, 2) or solvent (3)
*j*: index of iteration of number of crystallizers in a cascade
*k*^eff^: [g h^–1^ cm^–2^] effective crystallization rate constant, parameter of the SCM
*k*_α_: [g g^–1^ deg^–1^] calibration parameter of the polarimeter
*K*_M_: [g mL^–1^] kinetic parameter of enzymes
*K*_I_: [g mL^–1^] kinetic parameter of enzymes
*m*_*i*,C_: mass of the liquid phase in the crystallizer [g]
*m*_*i*,R_: mass of the liquid phase in the reactor [g]
*m*_max_^0^: maximum theoretical product mass [g]
*m*_*i*,C_^S^: mass of the solid phase in the crystallizer [g]
*m*_*i*_^S,0^: initial mass of the solid phase [g]
*m*_seeds_: mass of seeds [g]
*m*_tot_: total mass of the liquid phase [g]
*ṁ*: mass flow rate [g/h]
*M*_solvate_: molar mass of the solid solvate [g mol^–1^]
*M*_*i*_: molar mass of nonsolvated enantiomers [g mol^–1^]
*n*^eff^: effective order of crystallization kinetics [-]
*N*: number of particles [#]
pr: productivity [g h^–1^ L^–1^]
pu: purity [-]
*R*: radius of particles [cm]
*R*^0^: initial radius of particles [cm]
*r*_Rac_: rate of racemization [g h^–1^ cm^3^]
*S*: supersaturation [-]
*S*^0^: initial supersaturation [-]
*t*: time [h]
*t*_dead_: dead time or idle time during the PC process [h]
*t*_stop_: stop time, parameter of the SCM [h]
*T*: temperature [°C]
*T*_cryst_: crystallization temperature [°C]
*T*_sat_: saturation temperature [°C]
*V*_R_: volume of the reactor [cm^3^]
*V*_L_: volume of the crystallizer [cm^3^]
*V̇*: volumetric flow rate [cm^3^/min]
*w*^0^: initial mass fraction [g g ^–1^]
*w*: mass fraction [g g ^–1^]
*w*_sat_: saturation mass fraction [g g ^–1^]
*w*_sat_^0^: initial saturation mass fraction [g g ^–1^]
*X*: Cartesian coordinate [-]
*Y*: Cartesian coordinate [-]
yi: yield [-]
α: optical rotation [deg]
ρ_S_: density of the solid phase [g cm^–3^]
ρ_L_: density of the liquid phase [g cm^–3^]
ρ_water_: density of water [g cm^–3^]
*v*_max_: maximum rate achieved by the system [g h^–1^ mg enzyme^–1^]
τ: residence time [h]

## References

[ref1] De Camp W. H. The FDA perspective on the development of stereoisomers. Chirality 1989, 1, 2–6. 10.1002/chir.530010103. 2642032

[ref2] Stinson S. C. Chiral Pharmaceuticals. Chem. Eng. News 2001, 79, 79–97. 10.1021/cen-v079n040.p079.

[ref3] Myerson A. S. Handbook of Industrial Crystallization; Butterworth-Heinemann, 2002.

[ref4] Lorenz H.; Seidel-Morgenstern A. Processes To Separate Enantiomers. Angew. Chem., Int. Ed. 2014, 53, 1218–1250. 10.1002/anie.201302823. 24442686

[ref5] Juza M.; Mazzotti M.; Morbidelli M. Simulated moving-bed chromatography and its application to chirotechnology. Trends Biotechnol. 2000, 18, 108–118. 10.1016/s0167-7799(99)01419-5. 10675898

[ref6] Lorenz H.; Sheehan P.; Seidel-Morgenstern A. Coupling of simulated moving bed chromatography and fractional crystallisation for efficient enantioseparation. J. Chromatogr. A 2001, 908, 201–214. 10.1016/s0021-9673(00)00992-4. 11218123

[ref7] Wrzosek K.; García Rivera M. A.; Bettenbrock K.; Seidel-Morgenstern A. Racemization of undesired enantiomers: Immobilization of mandelate racemase and application in a fixed bed reactor. Biotechnol. J. 2016, 11, 453–463. 10.1002/biot.201500494. 26773335

[ref8] Xie R.; Chu L.-Y.; Deng J.-G. Membranes and membrane processes for chiral resolution. Chem. Soc. Rev. 2008, 37, 1243–1263. 10.1039/b713350b. 18497936

[ref9] Afonso C. A. M.; Crespo J. G. Recent Advances in Chiral Resolution through Membrane-Based Approaches. Angew. Chem., Int. Ed. 2004, 43, 5293–5295. 10.1002/anie.200460037. 15382202

[ref10] Majumder A.; Nagy Z. K. A comparative study of coupled preferential crystallizers for the efficient resolution of conglomerate-forming enantiomers. Pharmaceutics 2017, 9, 55 10.3390/pharmaceutics9040055. PMC5750661 29206148

[ref11] Coquerel G. Preferential crystallization. Top. Curr. Chem. 2007, 269, 1–51. 10.1007/128_2006_077. 23605348

[ref12] Köllges T.; Vetter T. Design and Performance Assessment of Continuous Crystallization Processes Resolving Racemic Conglomerates. Cryst. Growth Des. 2018, 18, 1686–1696. 10.1021/acs.cgd.7b01618.

[ref13] Jacques J.; Collet A.; Wilen S. H. Enantiomers, Racemates, and Resolutions; Krieger Pub. Co, 1994.

[ref14] Ishikawa H.; et al. Attrition-Enhanced Deracemization of Axially Chiral Nicotinamides. Eur. J. Org Chem. 2020, 1001–1005. 10.1002/ejoc.201901826.

[ref15] Xiouras C.; et al. Toward Continuous Deracemization via Racemic Crystal Transformation Monitored by in Situ Raman Spectroscopy. Cryst. Growth Des. 2019, 19, 5858–5868. 10.1021/acs.cgd.9b00867.

[ref16] Sögütoglu L.-C.; Steendam R. R. E.; Meekes H.; Vlieg E.; Rutjes F. P. J. T. Viedma ripening: a reliable crystallisation method to reach single chirality. Chem. Soc. Rev. 2015, 44, 6723–6732. 10.1039/c5cs00196j. 26165858

[ref17] Cameli F.; ter Horst J. H.; Steendam R. R. E.; Xiouras C.; Stefanidis G. D. On the Effect of Secondary Nucleation on Deracemization through Temperature Cycles. Chem.—Eur. J. 2020, 26, 1344–1354. 10.1002/chem.201904239. 31749171

[ref18] Intaraboonrod K.; Lerdwiriyanupap T.; Hoquante M.; Coquerel G.; Flood A. E. Temperature cycle induced deracemization. Mendeleev Commun. 2020, 30, 395–405. 10.1016/j.mencom.2020.07.002.

[ref19] Intaraboonrod K.; et al. Temperature Cycling Induced Deracemization of dl-Asparagine Monohydrate with Immobilized Amino Acid Racemase. Cryst. Growth Des. 2020, 21, 306–313. 10.1021/acs.cgd.0c01140.

[ref20] Levilain G.; Coquerel G. Pitfalls and rewards of preferential crystallization. CrystEngComm 2010, 12, 1983–1992. 10.1039/c001895c.

[ref21] Segel I. H. Enzyme Kinetics : Behavior and Analysis of Rapid Equilibrium and Steady State Enzyme Systems; Wiley Classics Library, 1975.

[ref22] Horn A.; Kumar S.; Liese A.; Kragl U. Reactions on Immobilized Biocatalysts. Handbook of Heterogeneous Catalysis; Wiley, 2008, pp. 3831–3865.

[ref23] Würges K.; Petruševska-Seebach K.; Elsner M. P.; Lütz S. Enzyme-assisted physicochemical enantioseparation processes-Part III: Overcoming yield limitations by dynamic kinetic resolution of asparagine via preferential crystallization and enzymatic racemization. Biotechnol. Bioeng. 2009, 104, 1235–1239. 10.1002/bit.22498. 19655380

[ref24] Carneiro T.; Wrzosek K.; Bettenbrock K.; Lorenz H.; Seidel-Morgenstern A. Immobilization of an amino acid racemase for application in crystallization-based chiral resolutions of asparagine monohydrate. Eng. Life Sci. 2020, 20, 550–561. 10.1002/elsc.202000029. 33304228 PMC7708953

[ref25] Oketani R.; Hoquante M.; Brandel C.; Cardinael P.; Coquerel G. Resolution of an Atropisomeric Naphthamide by Second-Order Asymmetric Transformation: A Highly Productive Technique. Org. Process Res. Dev. 2019, 23, 1197–1203. 10.1021/acs.oprd.9b00133.

[ref26] Harriehausen I.; Bollmann J.; Carneiro T.; Bettenbrock K.; Seidel-Morgenstern A. Characterization of an Immobilized Amino Acid Racemase for Potential Application in Enantioselective Chromatographic Resolution Processes. Catalysts 2021, 11, 726 10.3390/catal11060726.

[ref27] Yagishita F.; et al. Total Spontaneous Resolution by Deracemization of Isoindolinones. Angew. Chem., Int. Ed. 2012, 51, 13023–13025. 10.1002/anie.201205097. 23150288

[ref28] Steendam R. R. E.; Ter Horst J. H. Continuous Total Spontaneous Resolution. Cryst. Growth Des. 2017, 17, 4428–4436. 10.1021/acs.cgd.7b00761.

[ref29] Mullin J. W. Nucleation Crystallization; Butterworth-Heinemann, 2001, Chapter 5, pp. 181–215.

[ref30] Elsner M. P.; Ziomek G.; Seidel-Morgenstern A. Simultaneous preferential crystallization in a coupled, batch operation mode-Part I: Theoretical analysis and optimization. Chem. Eng. Sci. 2007, 62, 4760–4769. 10.1016/j.ces.2007.05.035.

[ref31] Elsner M. P.; Ziomek G.; Seidel-Morgenstern A. Efficient separation of enantiomers by preferential crystallization in two coupled vessels. AIChE J. 2009, 55, 640–649. 10.1002/aic.11719.

[ref32] Lorenz H.; Polenske D.; Seidel-Morgenstern A. Application of preferential crystallization to resolve racemic compounds in a hybrid process. Chirality 2006, 18, 828–840. 10.1002/chir.20327. 16917833

[ref33] Qamar S.; Galan K.; Peter Elsner M.; Hussain I.; Seidel-Morgenstern A. Theoretical investigation of simultaneous continuous preferential crystallization in a coupled mode. Chem. Eng. Sci. 2013, 98, 25–39. 10.1016/j.ces.2013.05.010.

[ref34] Galan K.; Eicke M. J.; Elsner M. P.; Lorenz H.; Seidel-Morgenstern A. Continuous preferential crystallization of chiral molecules in single and coupled mixed-suspension mixed-product-removal crystallizers. Cryst. Growth Des. 2015, 15, 1808–1818. 10.1021/cg501854g.

[ref35] Binev D.; Seidel-Morgenstern A.; Lorenz H. Continuous Separation of Isomers in Fluidized Bed Crystallizers. Cryst. Growth Des. 2016, 16, 1409–1419. 10.1021/acs.cgd.5b01513.

[ref36] Gänsch J.; et al. Continuous enantioselective crystallization of chiral compounds in coupled fluidized beds. Chem. Eng. J. 2021, 422, 129627 10.1016/j.cej.2021.129627.

[ref37] Temmel E.; Eicke M. J.; Cascella F.; Seidel-Morgenstern A.; Lorenz H. Resolution of Racemic Guaifenesin Applying a Coupled Preferential Crystallization-Selective Dissolution Process: Rational Process Development. Cryst. Growth Des. 2019, 19, 3148–3157. 10.1021/acs.cgd.8b01660. PMC7493326 32952448

[ref38] Lewis A. E.; Seckler M.; Kramer H. J. M.; van Rosmalen G. M. Industrial Crystallization: Fundamentals and Applications. Industrial Crystallization: Fundamentals and Applications; Cambridge University Press, 2015.

[ref39] Ramkrishna D. Population Balances : Theory and Applications to Particulate Systems in Engineering; Academic Press, 2000.

[ref40] Randolph A. D.; Larson M. A. Theory of Particulate Processes : Analysis and Techniques of Continuous Crystallization; Academic Press, 1988.

[ref41] Qamar S.; Elsner M. P.; Angelov I. A.; Warnecke G.; Seidel-Morgenstern A. A comparative study of high resolution schemes for solving population balances in crystallization. Comput. Chem. Eng. 2006, 30, 1119–1131. 10.1016/j.compchemeng.2006.02.012.

[ref42] Qamar S.; Mukhtar S.; Ali Q.; Seidel-Morgenstern A. A Gaussian quadrature method for solving batch crystallization models. AIChE J. 2011, 57, 149–159. 10.1002/aic.12264.

[ref43] Carneiro T.; Bhandari S.; Temmel E.; Lorenz H.; Seidel-Morgenstern A. Shortcut Model for Describing Isothermal Batch Preferential Crystallization of Conglomerates and Estimating the Productivity. Cryst. Growth Des. 2019, 19, 5189–5203. 10.1021/acs.cgd.9b00592. PMC7493424 32952449

[ref44] Petruševska-Seebach K. Overcoming Yield Limitations when Resolving Racemates by Combination of Crystallization and, or Chromatography with Racemization; Docupoint-Verl., 2012.

[ref45] MATLAB and Statistics Toolbox Release; Mathworks, 2017.computer program

[ref46] Chaplin M. F.; Martin F. .; Bucke C. Enzyme Technology; Cambridge University Press, 1990.

[ref47] Mateo C.; Palomo J. M.; Fernandez-Lorente G.; Guisan J. M.; Fernandez-Lafuente R. Improvement of enzyme activity, stability and selectivity via immobilization techniques. Enzyme Microb. Technol. 2007, 40, 1451–1463. 10.1016/j.enzmictec.2007.01.018.

[ref48] Rodrigues R. C.; Ortiz C.; Berenguer-Murcia Á.; Torres R.; Fernández-Lafuente R. Modifying enzyme activity and selectivity by immobilization. Chem. Soc. Rev. 2013, 42, 6290–6307. 10.1039/c2cs35231a. 23059445

